# Supplementary material for: Mutation status and prognostic value of KRAS and NRAS mutations in Moroccan colon cancer patients: A first report
Source: PLoS One. 2021 Mar 30;16(3):e0248522. doi: 10.1371/journal.pone.0248522 (PMC8009361; doi:10.1371/journal.pone.0248522)
Supplement: Attachment — Submitted filename: Response to Reviewers.docx [file pone.0248522.s001.docx]

**Response to reviewers**

**Title:** Mutation status and prognostic value of KRAS and NRAS mutations in Moroccan colon cancer patients: a first report

**Authors:** Fatima El agy^1,^ ^2*^, Sanae el Bardai^2,^ , Ihsane El Otmani^1,2,3^, Zineb Benbrahim^4^, Ibn Majdoub Hassani Karim^5^, Khalid Mazaz^5^, El Bachir Benjelloun^5^, Abdelmalek Ousadden^5^, Mohammed El Abkari^6^, Sidi Adil Ibrahimi^5^, Laila Chbani^1,2^.

**Author’s response to reviews:**

To the Editor, PLOS ONE

Dr. Hassan Ashktorab

Fes, Jeanery 2st, 2020

**Dear Editor,**

Thank you for your interest in our study. I am pleased to resubmit the revised version of our manuscript entitled “Mutation status and prognostic value of KRAS and NRAS mutations in Moroccan colon cancer patients: a first report” to be considered for publication in "PLOSONE”

We appreciate your comments that greatly improved the quality of the manuscript. In the following pages, we have addressed your remarks in a point by point format.

Looking forward to hearing from you.

Thanking you,

Yours sincerely,

Fatima El agy

Fatima El agy ( PhD)

Biomedical and translational research laboratory

Faculty of Medicine and Pharmacy,

Sidi Mohamed Ben Abdellah University, Fez, Morocco.

Tel: 0645572257

Email: [Fatima.elagy@usmba.ac.ma](mailto:Fatima.elagy@usmba.ac.ma)

**Journal requirements:**

1. Please ensure that your manuscript meets PLOS ONE's style requirements, including those for file naming:

**Response**: this point has been verified.

2. Our journal requires that methods are described in enough detail to allow suitably skilled investigators to fully replicate your study. If materials, methods, and protocols are well established, authors may cite articles where those protocols are described in detail, but the submission should include sufficient information to be understood independent of these references. Please revise your manuscript so that you methodology for BRAF molecular testing is briefly but sufficiently described.

In addition, please provide the primer sequences for all primers used.

Also, please change p-values of "0.000" to "<0.001" in your tables.

**Response**: the methodology of BRAF molecular testing has been briefly and sufficiently described “BRAF testing was performed for 200 patients using Sanger sequencing as described previously (KRAS and NRAS mutations testing). Briefly, DNA was amplified by PCR (Master Mix (2X) kits) according to the manufacturer’s protocols and using the following forward primer (5’-ACGAACGAGACTATCCTTTTAC-3’) and reverse primer (5’- CATTGAGTCGTCGTAGAGTCCC -3’). PCR products were purified using ExoSAP® kit according to the manufacturer’s protocols. The purified PCR products were sequenced using the direct sequencing with BigDye Terminator V3.1 Cycle Sequencing Kit (ABI Prism) and analyzed on Applied Biosystems 3500Dx Genetic Analyzer (Applied Biosystems).”

The primer sequences used for PCR have been added in methods section:

**Table 1**: Primer sequences used for PCR.

| Primer name | Primer sequence |
| --- | --- |
| KRAS-ex 2- F  KRAS-ex 2- R | 5’-GGTGGAGTATTTGATAGTGTA- 3’  5’-TGCATATTACTGGTGCAGACC- 3’ |
| KRAS-ex 3- F  KRAS-ex 3- R | 5’-AGTAAAAGGTGCACTGTAATAA-3’  5’-ATAATAAGCTGACATTAAGGAG-3’ |
| KRAS-ex 4- F  KRAS-ex 4- R | 5’-TGTTACTAATGACTGTGCTATAACTTTT-3’  5’-TATGCTATACTATACTAGGAAATAAAA-3’ |
| NRAS-ex2-F  NRAS-ex2-R | 5’-ATGACTGAGTACAAACTGGTGGTGGTTGGAGCA-3’  5’-CACTTTGTAGATGAATATGATCCCACCATAGAG-3’ |
| NRAS-ex3-F  NRAS-ex3-R | 5’-GATTCTTACAGAAAACAAGTGGTTA-3’  5’-CATTTGCGGATATTAACCTCTACAG-3’ |
| NRAS-ex4-F  NRAS-ex4-R | 5’-GGAGCAGATTAAGCGAG-3’  5’-TCAGCCAAGACCAGACAG-3’ |

The p-values of "0.000» have been changed to "<0.001" in all tables.

3. Please also provide additional information about the participant recruitment method and the demographic details of your participants. Please ensure you have provided sufficient details to replicate the analyses such as:
a) a description of any inclusion/exclusion criteria that were applied to participant recruitment,
b) a statement as to whether your sample can be considered representative of a larger population, and
c) a description of how participants were recruited.

**Response:** This point has been addressed in methods section. Medical charts were prospectively and retrospectively reviewed, and patients were recruited using the following selection criterion: (a) patients with histologically confirmed primary adenocarcinoma (b) all cases with pathologic I-IV stage colon cancer, (c) patients with prognostic information and who underwent surgical resection for CC tumor at the surgery department of Hassan II University Hospital. Patients were excluded if their records were incomplete, without histological confirmation of colon adenocarcinoma and if they had rectal cancer. Demographic and clinicopathological information (e.g. age, gender, tumor grade, tumor site histological subtype, disease stage, and numbers of examined regional lymph nodes…) and follow up data, were obtained from the patient’s medical records and pathology reports. Overall survival was defined as the time from the start of diagnosis until death or until the last follow-up.

4. Please provide additional details regarding participant consent.

In the ethics statement in the Methods and online submission information, please ensure that you have specified what type you obtained (for instance, written or verbal, and if verbal, how it was documented and witnessed). If your study included minors, state whether you obtained consent from parents or guardians. If the need for consent was waived by the ethics committee, please include this information

**Response**: This study protocol was reviewed and approved by Hassan II University Hospital Ethics Committee of FEZ, Morocco, under reference no. 13/18. All patients gave written informed consent before the start of the study.

5. We note that you have indicated that data from this study are available upon request. PLOS only allows data to be available upon request if there are legal or ethical restrictions on sharing data publicly. For more information on unacceptable data access restrictions, please see http://journals.plos.org/plosone/s/data-availability#loc-unacceptable-data-access-restrictions.

**Response:** this point has been addressed**.** Data Availability Statement: All relevant data are within the Manuscript.

6. Thank you for stating the following financial disclosure:

'The funders had no role in study design, data collection and analysis, decision to publish, or preparation of the manuscript.'

At this time, please address the following queries:

a. Please clarify the sources of funding (financial or material support) for your study. List the grants or organizations that supported your study, including funding received from your institution.
b. State what role the funders took in the study. If the funders had no role in your study, please state: “The funders had no role in study design, data collection and analysis, decision to publish, or preparation of the manuscript.”
c. If any authors received a salary from any of your funders, please state which authors and which funders.
d. If you did not receive any funding for this study, please state: “The authors received no specific funding for this work.”

**Response:** this point has been addressed. Funding: The authors received no specific funding for this work.

**Response to review comments:**

Reviewer #1:

This paper describes RAS mutations in Moroccan CRC patients (210). While the data is sound and confirms previous findings from other populations, this is the first of its kind in Morrocan patients. Changes need to be made to improve the manuscript. The Abstract is too long and the Discussion needs some revision.

**Response:** this point has been addressed in the manuscript**.**

**Abstract:** This study aimed to estimate the incidence of KRAS, NRAS, and BRAF mutations in the Moroccan population, and investigate the associations of KRAS and NRAS gene mutations with clinicopathological characteristics and their prognosis value. To achieve these objectives, we reviewed medical and pathology reports for 210 patients. RAS testing was investigated by Sanger sequencing and Pyrosequencing technology. BRAF (exon 15) status was analyzed by the Sanger method. The expression of MMR proteins was evaluated by Immunohistochemistry. KRAS and NRAS mutations were found in 36.7% and 2.9% of 210 patients, respectively. KRAS exon 2 mutations were identified in 76.5% of the cases. RAS-mutated colon cancers were significantly associated with female gender, presence of vascular invasion, classical adenocarcinoma, moderately differentiated tumors, advanced TNM stage III-IV, left colon site, higher incidence of distant metastases at the time of diagnostic, microsatellite stable phenotype, lower number of total lymph nodes, and higher means of positive lymph nodes and lymph node ratio. KRAS exon 2-mutated colon cancers, compared with KRAS wild-type colon cancers were associated with the same clinicopathological features of RAS-mutated colon cancers. NRAS-mutated patients were associated with lower total lymph node rate and the presence of positive lymph node. Rare RAS-mutated tumors, compared with wild-type tumors were more frequently moderately differentiated and associated with lower lymph node rate. We found that KRAS codon 13-mutated, tumors compared to codon 12-mutated tumors were significantly correlated with a higher death cases number, a lower rate of positive lymph, lower follow-up time, and poor overall survival. Our findings show that KRAS and NRAS mutations have distinct clinicopathological features. KRAS codon 13-mutated status was the worst predictor of prognosis at all stages in our population.

Why is it that an unmethylated control used in Purosequencing, this study is not about methylation?

**Response:** this positive control has been designed by the supplier for use in both methylation and PCR testing.

The authors need to explain how was MSI status established, it is NOT in Methods section?

**Response:** The mismatch repair tumor status (MSS or MSI) was established by immunohistochemistry (IHC) to detect the intact or the loss expression of the MMR proteins (MLH1, PMS2, MSH2, and MSH6). The IHC study was assessed on unstained formalin-fixed paraffin-embedded (FFPE) tumor tissue sections of 4 *μ*m thickness, on the automated immunostainer Ventana Benchmark ULTRA. We have employed monoclonal antibodies specific for each MMR protein, MLH1 (G168-728/CELL MARQUE), MSH2 (G219-1129/CELL MARQUE), MSH6 (44/CELL MARQUE), and PMS2 (MRQ-28/CELL MARQUE). Adjacent normal tissue (lymphocytes or normal glandular cells) was used as an internal control for positive staining.

In Methods, they stated they compared stges I-II vs II-IV, I think you mean I-II vs. III-IV, Pease correct.
**Response:** This point has been corrected.

Please adjust write up to show that mutant Kras generally needed LESS LN to be examined to get positve ones.

**Response:** Furthermore the presence of positive lymph node was significantly associated with KRAS mutated tumors.

For NRAS mutations, is it 2-8 or 2-9%, you are putting different numbers in different places?

**Response**: This point has been corrected in the text; it is 2.9%.

Would suggest to drop RAS muataions part from text and directly just state KRAS and NRAS.

**Response**: this point has been addressed in the text.

In tables, please bolden and underline SIGNIFICANT p VALUES.

**Response**: this point has been addressed in all tables.

| **Characteristics** | **RAS Wild-type** | **RAS Mutants** | **p-value** |
| --- | --- | --- | --- |
| **Age:**  Mean (±SD)  ≤55  ≥55 | 55.3 (±14.3)  57 (44.9%)  70 (55.1%) | 55.9 (±15.7)  35 (42.2%)  48 (57.8%) | 0.7  0.1 |
| **Gender**  Female  Male | 49 (38.6%)  78 (61.4%) | 48 (57.8%)  35 (42.2%) | **0.003** |
| **Tumor site**  Right colon  Left colon | 48(37.8%)  79 (62.2%) | 19 (22.9%)  64 (77.1%) | **0.009** |
| **Histologic subtype:**  Adenocarcinoma  Mucinous adenocarcinoma  Others | 107 (84.3%)  10 (7.9%)  10(7.9%) | 65 (78.3%)  16 (19.3%)  2 (2.4%) | **0.01** |
| **Histologic grade :**  Well  moderate  Poor | 58 (45.7%)  54 (42.5%)  15 (11.8%) | 34 (41.0%)  45 (54.2%)  4 (4.8%) | **0.04** |
| **Vascular invasion:**  Yes  No | 15 (11.8%)  112 (88.2%) | 22 (26.5%)  61(73.5%) | **0.006** |
| **perineural invasion:**  Yes  No | 14 (11.0%)  113 (89.0%) | 8 (10.1%)  71(89.9%) | 0.1 |
| Distant metastases (M)  M0  M1 | 82 (64.6%)  45 (35.4%) | 45 (54.2%)  38 (45.8%) | **0.03** |
| Number of metastases:  1  ≥2 | 31 (73.8%)  11 (26.2%) | 20 (60.6%)  13 (39.4%) | **0.04** |
| Number of removed lymph nodes, mean ± SD  <12  ≥12 | 20.43 ±9.37  18 (14.2%)  109 (85.8%) | 14.83 ±9.19  28 (38.9%)  44 (61.1%) | **<0.001**  **<0.001** |
| Positive lymph node:  Mean (± SD)  Presence  Absence  Lymph node ratio, mean ± SD | 1.34 (±3.70)  29 (22.8%)  98 (77.2%)  0.07 ±0.18 | 2.44 (±3.4)1  35 (48.6%)  37 (51.4%)  0.32 ±0.55 | **0.03**  **<0.001**  **<0.001** |
| Disease stages  I-II  III-IV | 57 (44.9%)  70 (55.1%) | 27 (32.5%)  56 (67.5%) | **0.02** |
| MSI status  MSI  MSS | 15 (19.5%)  62 (80.5%) | 2 (5.0%)  38 (95.0%) | **0.02** |
| BRAF mutation: | - | - | **-** |
| **follow-up time (months):**  **Mean**  SD | 50.08  ±34.19 | 41.69  ±34.63 | **0.04** |
| **Recurrence**  (+)  (−) | 36 (28.3%)  91 (71.7%) | 39 (47.0%)  44 (53.0%) | **0.003** |
| **Mortality**  Death cases  Censored cases | 26(20.5%)  101 (79.5%) | 27 (32.9%)  55 (67.1%) | **0.01** |
|  |  |  |  |
|  |  |  |  |

| Characteristics | KRAS mutant | KRAS wild type | p | NRAS  Mutant | NRAS wild type | p | Rare mutations | wild-type  status | p |
| --- | --- | --- | --- | --- | --- | --- | --- | --- | --- |
| **Age**  Mean ((±SD)  ≤55  >55 | 55.8 ± 16.0  33 (42.9%)  44 (57.1% | 55.3 ±14.3  59 (44.4%)  74 (55.6%) | 0.7  0.1 | 56.5 ±13.0  2 (33.3%)  4 (66.7%) | 55.5 ±14.9  90 (44.1%)  114 (55.9%) | 0.8  0.4 | 50.2 ±16.0  7 (46.7%)  8 (53.3%) | 55.8 ±14.7  85 (43.6%)  110 (56.4%) | 0.1  0.5 |
| **Gender**  Female  Male | 45(58.4%)  32(41.6%) | 52 (39.1%)  81(60.9%) | **0.003** | 3 (50.0%)  3 (50.0%) | 94 (46.1%)  110 (53.9%) | 0.4 | 7 (46.7%)  8 (53.3%) | 90 (46.2%)  105 (53.8%) | 0.5 |
| **Tumor site**  Right colon  Left colon | 18 (23.4%)  59 (76.6%) | 49(36.8%)  84 (63.2%) | **0.01** | 1 (16.7%)  5 (83.3%) | 66 (32.4%)  138 (67.6%) | 0.2 | 2 (13.3%)  13 (86.7%) | 65 (33.3%)  130 (66.7%) | 0.06 |
| **Histologic subtype**  Adenocarcinoma  Mucinous adenocarcinoma  Others | 60 (77.9%)  15(19.5%)  2 (2.6%) | 112(84.2%)  11 (8.3%)  10 (7.5%) | **0.02** | 5 (83.3%)  1(16.7%)  0 (0.0%) | 167(81.9%)  25 (12.3%)  12(5.9%) | 0.5 | 12 (80.0%)  3(20.0%)  0 (0.0%) | 160 (82.1%)  23 (11.8%)  12 (6.2%) | 0.4 |
| **Histologic grade**  Well  Moderate  poor | 33 (42.9%)  40 (51.9%)  4 (5.2%) | 59 (44.4%)  59(44.4%)  15 (11.3%) | 0.07 | 1(16.7%)  5 (83.3%)  0 (0.0%) | 91 (44.6%)  94 (46.1%)  19 (9.3%) | 0.1 | 2 (13.3%)  13 (86.7%)  0 (0.0%) | 90 (46.2%)  86 (44.1%)  19 (9.7%) | **0.006** |
| **vascular invasion**  Presence  Absence | 20 (26.0%)  57(74.0%) | 17(12.8%)  116(87.2%) | **0.04** | 0 (0.0%)  6 (100.0%) | 26 (12.7%)  178 (87.3%) | 0.4 | 0 (0%)  15 (100%) | 26 (13.3%)  169 (86.7%) | 0.09 |
| **Perineural invasion**  Presence  Absence | 8 (11.0%)  65 (89.0%) | 14(10.5%)  119(89.5%) | 0.1 | 2 (33.3%)  4 (66.7%) | 35 (17.2%)  169 (82.8%) | 0.5 | 4 (26.7%)  11 (73.3%) | 33 (16.9%)  162 (83.1%) | 0.1 |
| **Distant metastases (M)**  M0  M1 | 42 (54.4%)  35 (45.5%) | 85 (63.9%)  48 (36.1%) | **0.04** | 3 (50.0%)  3 (50.0%) | 83 (65.4%)  44 (34.6%) | 0.3 | 8 (53.3%)  7 (46.7%) | 119 (61.0%)  76 (39.0%) | 0.3 |
| **Number of metastases:**  1  ≥2 | 18 (62.1%)  11 (37.9%) | 33(76.7%)  10(23.3%) | 0.1 | 3 (100.0%)  0 (0.0%) | 49 (70.0%)  21 (30.0%) | 0.3 | 3 (40.0%)  2 (60.0%) | 48 (71.6%)  19 (28.4%) | 0.4 |
| **Removed lymph nodes:**  mean ± SD  <12  ≥12 | 15.0 ±9.4  25 (37.9%)  41 (62.1%) | 20.0 ±9.3  21 (15.8%)  112(84.2%) | **<0.001**  **<0.001** | 12.2 ±5.8  3 (50.0%)  3 (50.0%) | 18.6 ±9.7  43 (22.3%)  150 (77.7%) | **0.04**  0.1 | 12.3 ±7.0  6 (46.2%)  7 (53.8%) | 18.82 ±9.7  146 (78.5%)  40 (21.5%) | **0.007**  **0.04** |
| **Positive lymph node**:  Mean (± SD)  Presence  Absence  Lymph node ratio, mean ± SD | 2.4 ±3.4  30 (45.5%)  36 (54.4%)  0.3 ±0.5 | 1.4 ±3.7  34 (25.6%)  99 (74.4%)  0.1 ±0.1 | 0.07  **0.003**  **<0.001** | 0.83 ±0.4  5 (83.3%)  1 (16.7%)  0.4 ±0.46 | 1.7 ±3.6  59 (30.6%)  134 (69.4%)  0.2 ±0.4 | 0.2  **0.01**  0.1 | 2.3 ±3.0  7 (53.8%)  6 (46.2%)  0.3 ±0.4 | 1.7 ±3.6  57 (30.6%)  129 (69.4%)  0.2 ±0.4 | 0.9  0.08  0.1 |
| Disease stages  I-II  III-IV | 26 (33.8%)  51 (66.2%) | 58(43.6%)  75 (56.4%) | **0.04** | 1 (16.7%)  5 (83.3%) | 83 (40.7%)  121 (59.3%) | 0.1 | 5 (33.3%)  10 (66.7%) | 79 (40.5%)  116 (59.5%) | 0.3 |
| BRAF mutation: | - | - | - | - | - | - | - | - | - |
| **Phenotype MSI:**  MSI  MSS | 2 (5.1%)  37 (94.9%) | 15 (19.2%)  63 (80.8%) | **0.03** | 0 (0.0%)  1 (100.0%) | 17 (14.7%)  99 (85.3%) | 0.6 | 0 (0.0%)  7 (100.0%) | 17 (15.5%)  93 (84.5%) | 0.3 |
| **Follow-up time (months)**  **Mean**  **SD** ± | 41.4  ±35.3 | 49.8  ±33.8 | 0.09 | 44.5 ±27.0 | 46.8  ±34.7 | 0.6 | 38.1  ±30.7 | 47.7  ±34.7 | 0.2 |
| **Recurrence**  (+)  (−) | 37 (48.1%)  40 (51.9%) | 38 (28.6%)  95 (71.4%) | **0.02** | 2 (33.3%)  4 (66.7%) | 36 (28.3%)  91 (71.7%) | 0.5 | 7(46.7%)  8 (53.3%) | 68 (34.9%)  127 (65.1%) | 0.2 |
| **Mortality**  Death cases  Censored cases | 26 (34.2%)  50 (65.8%) | 27 (20.3%)  106(79.7%) | **0.02** | 1(16.7%)  5 (83.3%) | 73 (35.8%)  131(64.2%) | 0.6 | 3 (20.0%)  12 (80.0%) | 50 (25.8%)  144 (74.2%) | 0.4 |

| Characteristics | KRAS codon 12 mutant | KRAS codon 12 wild type | p | Kras codon 13 mutants | KRAS codon 13 wild type | p | KRAS codon 12  mutants | KRAS codon 13 mutants | p |
| --- | --- | --- | --- | --- | --- | --- | --- | --- | --- |
| **Age**  Mean ((±SD)  ≤55  >55 | 57.2 ± 14.0  22 (42.3%)  30 (57.7% | 55.0 ±15.1  70 (44.3%)  88 (55.7%) | 0.3  0.4 | 57.3±20.1  6 (37.5%)  10 (62.5%) | 55.4±14.3  86 (44.3%)  108 (55.7%) | 0.6  0.1 | 57.2 ± 14.0  22 (42.3%)  30 (57.7%) | 57.3±20.1  6 (37.5%)  10 (62.5%) | 0.9  0.2 |
| **Gender**  Female  Male | 31(59.6%)  21(40.4%) | 66 (41.8%)  92 (58.2%) | **0.01** | 10 (62.5%)  6 (37.5%) | 87 (44.8%)  107 (55.2%) | 0.08 | 31(59.6%)  21(40.4%) | 10 (62.5%)  6 (37.5%) | 0.2 |
| **Tumor site**  Right colon  Left colon | 14 (26.9%)  38(73.1%) | 53(33.5%)  105(66.5%) | 0.2 | 3 (18.8%)  13 (81.3%) | 64 (33.0%)  130 (67.0%) | 0.1 | 14 (26.9%)  38(73.1%) | 3 (18.8%)  13 (81.3%) | 0.09 |
| **Histologic subtype**  Adenocarcinoma  Mucinous adenocarcinoma  Others | 43 (82.7%)  7 (13.5%)  2 (3.8%) | 133(84.2%)  15 (9.5%)  10 (6.3%) | **0.02** | 14(87.5%)  2(12.5%)  0 (0.0%) | 158 (81.4%)  24 (12.4%)  12 (6.2%) | 0.1 | 43 (82.7%)  7 (13.5%)  2 (3.8%) | 14(87.5%)  2(12.5%)  0 (0.0%) | 0.1 |
| **Histologic grade**  Well  Moderate  poor | 24 (46.2%)  24 (46.2%)  4 (7.7%) | 68 (43.0%)  75 (47.5%)  15 (9.5%) | 0.8 | 8(50.0%)  8 (50.0%)  0 (0.0%) | 84 (43.3%)  91 (46.9%)  19 (9.8%) | 0.1 | 24 (46.2%)  24 (46.2%)  4 (7.7%) | 8(50.0%)  8 (50.0%)  0 (0.0%) | **0.1** |
| **Vascular invasion**  Presence  Absence | 15 (28.8%)  37 (71.2%) | 22 (13.9%)  136(86.1%) | **0.01** | 3 (18.8%)  13(81.3%) | 34 (17.5%)  160 (82.5%) | 0.1 | 8 (16.3%)  41 (83.7%) | 2 (12.5%)  14 (87.5%) | 0.1 |
| **Perineural invasion**  Presence  Absence | 7 (14.3%)  42 (85.7%) | 15 (9.6%)  142(90.4%) | 0.2 | 0 (0.0%)  16 (100.0%) | 26 (13.4%)  168 (86.6%) | 0.1 | 7 (14.3%)  42 (85.7%) | 0 (0.0%)  16 (100.0%) | 0.1 |
| **Distant metastases (M)**  M0  M1 | 29 (55.8%)  23 (44.2%) | 98 (62.0%)  60 (38.0%) | 0.2 | 8 (50.0%)  8 (50.0%) | 119 (61.3%)  75 (38.7%) | 0.1 | 29 (55.8%)  23 (44.2% | 8 (50.0%)  8 (50.0%) | 0.2 |
| **Number of metastases:**  1  ≥2 | 15 (71.4%)  6 (28.6%) | 36 (70.6%)  15 (29.4%) | 0.5 | 2 (33.3%)  4 (66.7%) | 49 (73.1%)  18 (26.9%) | 0.1 | 15 (71.4%)  6 (28.6%) | 2 (33.3%)  4 (66.7%) | 0.08 |
| **Removed lymph nodes:**  mean ± SD  <12  ≥12 | 15.6±10.1  16 (36.4%)  28 (63.6%) | 20.4 ±9.3  30 (19.4%)  125(80.6%) | **<0.001**  **0.01** | 14.6 ±7.9  6 (40.0%)  9 (60.0%) | 18.7 ±9.7  40(21.7%)  144 (78.3%) | **0.05**  **0.05** | 15.6±10.1  16 (36.4%)  28 (63.6%) | 14.6 ±7.9  6 (40.0%)  9 (60.0%) | 0.7  0.2 |
| **Positive lymph node**:  Mean (± SD)  Presence  Absence  Lymph node ratio, mean ± SD | 2.9 ±3.7  22 (50.0%)  22 (50.0%)  0.4±0.6 | 1.4 ±3.5  42 (27.1%)  113(72.9%)  0.1 ±0.2 | **0.02**  **0.004**  **<0.001** | 1.3 ±2.4  6 (40.0%)  9 (60.0%)  0.2 ±0.3 | 1.7 ±3.7  58 (31.5%)  126 (68.5%)  0.1 ±0.4 | **0.5**  0.1  0.9 | 2.9 ±3.7  22 (50.0%)  22 (50.0%)  0.4±0.6 | 1.3 ±2.4  6 (40.0%)  9 (60.0%)  0.2 ±0.3 | **0.01**  **0.1**  **0.02** |
| Disease stages  I-II  III-IV | 15 (28.8%)  37 (71.2%) | 69 (43.7%)  89 (56.3%) | **0.04** | 7 (43.8%)  9 (56.3%) | 77 (39.7%)  117 (60.3%) | 0.1 | 15 (28.8%)  37 (71.2%) | 7 (43.8%)  9 (56.3%) | 0.2 |
| BRAF mutation: | - | - | - | - | - | - | - | - | - |
| **Phenotype MSI:**  MSI  MSS | 2 (8.0%)  23 (92.0%) | 15 (16.3%)  77 (83.7%) | 0.2 | 0 (0.0%)  8 (100.0%) | 17 (15.6%)  92 (84.4%) | 0.2 |  |  |  |
| **Follow-up time (months)**  **Mean**  **SD** ± | 46.6  ±37.8 | 46.7  ±33.5 | 0.9 | 29.0  ±23.3 | 48.0  ±34.9 | **0.006** | 46.6  ±37.8 | 29.0  ±23.3 | **<0.001** |
| **Recurrence**  (+)  (−) | 25 (48.1%)  27 (51.9%) | 50 (31.6%)  108(68.4%) | **0.02** | 7 (43.8%)  9 (56.3%) | 68(35.1%)  126 (64.9%) | 0.1 | 25 (48.1%)  27 (51.9%) | 7 (43.8%)  9 (56.3%) | 0.2 |
| **Mortality**  Death cases  Censored cases | 15 (28.8%)  37 (71.2%) | 38 (24.1%)  120(75.9%) | 0.2 | 9(56.3%)  7 (43.8%) | 44(22.8%)  149 (77.2%) | **0.003** | 15 (28.8%)  37 (71.2%) | 9(56.3%)  7 (43.8%) | **0.03** |

| Variables | Univariate analysis  Mean OS months (95% CI) P value | Multivariate analysis  Hazard ratio (95% CI) P value |
| --- | --- | --- |
| Age (yr)  ≥55  <55 | 95.52(84.85-106.18) 0.9  96.03(83.19-108.88) |  |
| Gender  Female  Male | 91.84 (78.98-104.70) 0.4  98.09 (87.26-108.92) |  |
| Tumor site  Right side  Left side | 93.75 (79.58-107.92) 0.8  90.34 (81.79-98.90) |  |
| Histologic subtype  Adenocarcinoma  Mucinous adenocarcinoma  Others | 95.42 (86.08-104.76)  94.11 (74.50-113.73) 0. 3  61.33 (37.33-85.33) |  |
| Histologic grad  Well  Moderate  Poor | 96.23 (84.53-107.94) 0.6  96.65 (84.05-109.25)  73.45 (51.38-95.52) |  |
| Distant metastases  M0  M1 | 105.10 (96.10-114.10) **0.001**  75.33 (59.12-91.54) | 0.34 (0.61-2.77) 0.4 |
| Number of metastases:  1  ≥2 | 50.12 ( 34.71-65.54) 0.1  84.45 (56.81-112.09) | 1.26 (0.05-1.05) 0.05 |
| Tumor stage  I-II  III-IV | 109.41(99.52-119.51) **0.002**  84.63 (72.57-96.69) | 1.98 (0.00-0.21) **0.003** |
| TLN  ≥12  <12 | 102.26 (93.41-111.10) **0.01**  72.51 (57.02-88.01) | 0.66 (0.29-3.32) 0.9 |
| Positive LN  Presence  Absence | 80.54 (66.88-94.19) **0.04**  102.09 (92.71-111.47 | 0.31 (0.46-4.15) 0.5 |
| Perineural invasion  Yes  No | 91.56 (77.95-105.18) 0.2  94.97 (86.34-103.60) |  |
| Vascular invasion  Yes  No | 102.23 (90.81-113.66) **0.02**  92.25 (83.25-101.25) | 1.93 (0.20-20.78) 0.5 |
| MSI status  MSI  MSS | 109.71 (99.85-119.58) **0.001**  74.08 (68.00-80.16) | 0.11 (0.06-1.04) 0.05 |
| Full RAS status  Wild-type  Mutant | 104.29 (94.35-114.02) **0.006**  78.18 (65.84-90.52) | 0.33 (0.00-4.50) 0.9 |
| KRAS status  Wild-type  Mutant | 104.14 (94.48-113.80) **0.003**  76.81 (63.89-89.74) | 0.29 (0.00-3.01) 0.9 |
| NRAS status  Wild-type  Mutant | 95.68 (87.36-104.01) 0.6  73.00 (48.99-97.00) |  |
| KRAS exons 2 codon:  codon 12/13 WT  codon 12 mutant  codon 13 mutant | 103.75 (94.32-113.17) **<0.001**  84.56 (69.29-99.82)  40.64 (23.02-58.27) | 0.35 (0.82-14.08) **0.01** |

The paper also uses many unusual abbreviations amd has many typos and grammatical errors. Please review completely.

**Response:** This point has been checked.

Reviewer #2:

There is a slight redundancy in the Introduction such as “Ras-family G-proteins transduce signals in growth factor signals, such as EGF receptor (EGFR) signaling pathway (1). I suggest you strike out “signals” that appears before “in growth factor”.

**Response:** Ras-family G-proteins transduce in growth factor signals, such as EGF receptor (EGFR) signaling pathway.

The citation numbers need a single space separation from the words. Make space between the last words before cited reference numbers in your text.

**Response:** this point has been addressed.

Your statement that “KRAS mutations occur in 45% of colorectal cancer (CRC) and NRAS in only 5-8%(6)” should be clarified to indicate whether this is applicable to the Moroccan patients or is referring to the world-wide CRC patients.

**Response:** Numerous studies have reported that KRAS and NRAS mutations occur respectively in 45% and 5-8% of worldwide colorectal cancer patients (6).

-Essentially, the authors of this article are attempting to mimic the work of the French authors in Molecular Cancer Genetics Platform of Poitiers (French), who have published their results about the clinicopathological characteristics of the RAS mutations in a cohort of over 1500 colorectal cancer patients. Was the French study done exclusively with French CRC patients?

**Response:** Recently, the Molecular Cancer Genetics Platform of Poitiers (French) has published interesting results about the clinicopathological features of every KRAS and NRAS mutation in a cohort of 1735 French colorectal cancer patients, enrolled from 28 hospital molecular genetics platforms throughout France (15).

-Table 6. Any reason why the multivariate analysis was not done in some of the metastatic tumors in Table 6, where the molecular and clinical variables associated with overall survivals in the 210 colon cancer patients were shown?

**Response:** The multivariate analysis was performed; we just forgot to present the results in table 6. 0.34 (0.61-2.77)	0.4

-In the body of the text, especially in the Results section, it was quite difficult to follow the different mutational hotspots from gene site to gene site, it would have been better to display the most common types in tabular format for quicker views and understanding of the cardinal gene alterations.

**Response:** Table 3: Mutational status of patients with CC by genes.

| **Gene alterations** | **Number (%)** |
| --- | --- |
| **KRAS** (n=210) **:**  Wildtype (n=133)  Mutated (n=77) | 66.3%  36.7% |
| **NRAS** (n=210) **:**  Wildtype (n=204)  Mutated (n=6) | 97.1%  2.9% |
| **BRAF** (n=200) **:**  Wildtype  Mutated | 100%  0% |

-The impetus to investigate the WT and mutated RAS gene sites was that mutations in KRAS outside exon 2 (KRAS exons 3,4) and in other downstream effectors of the EGFR signaling pathway (like NRAS) were published to be associated with resistance to anti-EGFR monoclonal antibodies that were used as a standard regimen for CRC patients – meaning that the two Anti-EGFR monoclonal antibodies, panitumumab and cetuximab, used to treat CRC patients with KRAS and NRAS mutations, were not effective. Therefore, the policy was to successfully treat CRC patients, the full RAS WT status had to be confirmed in all mCRC patients before initiating treatment. The question is: Does this study conclusively resolve that issue? If so, that must be clearly stated in the manuscript.

**Response:** this point has been reported in methods section; Since 2016, we have started testing complete RAS status systematically, for every metastasis colon cancer patient when considered for anti-EGFR therapy.

-Compared to NRAS wild-type patients, “NRAS-mutated patients seem to be exhibiting lower total lymph node rate, and were associated with higher lymph node metastasis number”. This statement is confusing. It could be revised to make the right statement without seemingly contradicting itself.

**Response:** Compared to NRAS wild- type patients, NRAS-mutated patients, were more likely to exhibit lower total lymph node rate (P=0.04). This subgroup of tumors was also marked by the presence of positive lymph node (83.3%, P=0.01).

-Among RAS mutations, KRAS codon 13 mutations seemed to have a poor prognostic value, which may negatively impinge on overall survival of all the stages of CRC patients. Is that a correct statement?

**Response:** this statement has been reviewed; KRAS codon 13-mutated status was the worst predictor of prognosis at all stages in our population.

All revisions have been highlighted in the marked copy of the manuscript.
